# Real-world outcomes of regorafenib combined with immune checkpoint inhibitors in patients with advanced or metastatic microsatellite stable colorectal cancer: A multicenter study

**DOI:** 10.1007/s00262-021-03083-3

**Published:** 2021-10-24

**Authors:** Kaili Yang, Lu Han, Shikai Wu, Xiujuan Qu, Qin Li, Chuanhua Zhao, Jing Zhou, Xuan Jin, Yusheng Wang, Dong Yan, Zhiqiang Cheng, Yuwei Hua, Yan Zhang, Yang Ge, Jinghua Sun, Wei Deng, Lin Zhao, Yunbo Zhao

**Affiliations:** 1 Department of Medical Oncology, Peking Union Medical College Hospital, Chinese Academy of Medical Sciences and Peking Union Medical College, Beijing, 100032 China; 2 Department of Medical Oncology, The General Hospital of People’s Liberation Army, Beijing, 100853 China; 3 Department of Medical Oncology, Peking University First Hospital, Beijing, 100034 China; 4 Department of Medical Oncology, The First Hospital of China Medical University, Shenyang, 110001 China; 5 Department of Oncology, Beijing Friendship Hospital, Capital Medical University, Beijing, 100050 China; 6 Department of Oncology, 307 Hospital of People’s Liberation Army, Beijing, 100071 China; 7 Department of Gastrointestinal Surgery, Peking University People’s Hospital, Beijing, 100044 China; 8 Department of Digestive System, Shanxi Cancer Hospital, Taiyuan, 030013 Shanxi China; 9 Cancer Center, Beijing Luhe Hospital, Capital Medical University, Beijing, 101149 China; 10 Department of Oncology of Integrative Chinese and Western Medicine, China-Japan Friendship Hospital, Beijing, 100029 China; 11 Department of General Surgery, Xuanwu Hospital, Capital Medical University, Beijing, 100053 China; 12 Department of Medical Oncology, Beijing Chaoyang Hospital, Capital Medical University, Beijing, 100021 China; 13 Department of Oncology, The Second Hospital of Dalian Medical University, Dalian, 116044 China; 14 Department of General Surgery, Beijing Friendship Hospital, Capital Medical University, Beijing, 100050 China; 15 Department of Oncology, Beijing Hospital, National Center of Gerontology, Beijing, 100730 China

**Keywords:** Colorectal cancer, Combination therapy, Immune checkpoint inhibitor, Regorafenib, Real world

## Abstract

**Background:**

Treatment strategies are limited for patients with chemotherapy refractory microsatellite stable (MSS) colorectal cancer. We aim to evaluate the efficacy and safety of immune checkpoint inhibitors (ICIs) combined with regorafenib in this population in routine clinical practice.

**Methods:**

We retrospectively analyzed patients with advanced or metastatic colorectal cancer who received at least one dose of ICIs combined with regorafenib in 14 Chinese medical centers. The primary outcome was objective response rate (ORR). This study was registered at ClinicalTrials.gov on February 2020 (NCT04771715).

**Results:**

Eighty-four patients received ICIs combined with regorafenib from January 2019 to January 2021. Most patients (91%) received two or more systemic treatment lines before the study treatment. Seventy-six patients (90%) had confirmed MSS status. At a median follow-up of 5.5 months, four patients achieved partial response (5%) and 37 patients achieved stable disease (45%) as the best response. The median progression-free survival (PFS) was 3.1 months, and the median overall survival was 17.3 months. Eleven patients (13%) remained progression-free for more than 6 months. Baseline liver metastasis (HR 1.98, 95%CI 1.07–3.69, *P* = 0.03) and neutrophil–lymphocyte ratio (NLR) of ≥ 1.5 (HR 2.83, 95%CI 1.00–7.98, *P* = 0.05) were associated with shorter PFS in multivariate analysis. Grade 3 or higher treatment-related adverse events (TRAEs) occurred in 16 patients (19%).

**Conclusion:**

The combination of ICIs with regorafenib can be a valuable treatment option for a proportion of patients with chemotherapy refractory MSS colorectal cancer. Patients with no liver metastasis and a low NLR at baseline may derive most benefit from this strategy.

**Supplementary Information:**

The online version contains supplementary material available at 10.1007/s00262-021-03083-3.

## Introduction

Colorectal cancer is the third most common cancer in the world with ascending incidence and mortality over the last decade [1]. Although the integration of targeted therapy into clinical practice has significantly increased the overall survival of patients with metastatic colorectal cancer (mCRC), treatment options after disease progression to standard of care are limited with modest survival benefit, and the long-term survival for chemotherapy refractory mCRC patients remains poor [2–4]. There is an unmet need for effective treatment strategies for these patients.

Immune checkpoint inhibitors (ICIs) have demonstrated promising efficacy in patients with microsatellite instability-high (MSI-H) or mismatch repair-deficient (dMMR) mCRC [5–7]. However, MSI-H/dMMR tumors only account for 2–4% of the total mCRC cases [8]. Most colorectal cancer patients have a microsatellite stable (MSS) or mismatch repair proficient (pMMR) status and obtain little benefit from ICIs [9, 10]. The difference of the tumor immune microenvironment between the two subtypes may account for the distinct response [11–13]. Thus, it is reasonable to apply combination strategies to modulate the microenvironment of MSS colorectal cancer and therefore exploit the benefit of ICIs. Regorafenib is a small molecule multi-kinase inhibitor that has been approved for treating chemotherapy refractory mCRC [14]. Besides its antiangiogenic effect, preclinical studies have demonstrated that regorafenib could modulate macrophage polarization and inhibit the expression of immunosuppressive molecules, which restored the immunosuppressive tumor microenvironment and synergistically enhanced the efficacy of ICIs [14–16]. A phase Ib trial has reported that the combination of nivolumab with regorafenib achieved an objective response rate of 33% in patients with MSS/pMMR mCRC [17]. Meanwhile, in a recent phase II trial investigating avelumab combined with regorafenib, patients only achieved stable disease as the best response [18].

Therefore, the combination of ICIs with regorafenib may be a promising treatment strategy for patients with MSS/pMMR mCRC. However, this strategy has been only applied in phase I or II trials with small sample sizes, where patients are usually hyperselected [19, 20]. Whether this combination is effective for the heavily pretreated patients with multiple comorbidities in routine clinical practice remains unknown. To elucidate these issues, we conducted this retrospective study to evaluate the efficacy and safety of regorafenib combined with ICIs for patients with advanced or metastatic MSS colorectal cancer in the real world.

## Methods

This retrospective study was conducted in 14 Chinese medical centers according to the Strengthening the Reporting of Observational Studies in Epidemiology (STROBE) reporting guideline [21]. The study was approved by the institutional review board of all participating centers and was registered at ClinicalTrials.gov (NCT04771715) on February 2021. Patients’ consents for participation and publication were not required because of the retrospective design and the deidentified data of this study.

### Patients

We reviewed electronic medical records to identify patients with advanced or metastatic colorectal cancer who received at least one dose of ICIs combined with regorafenib from January 2019 to January 2021. The types of ICIs, treatment doses and schedules were determined per investigator’s decision. Previous exposure to ICIs or regorafenib was acceptable. Patients with confirmed MSI-H/dMMR status were excluded.

### Outcome

The primary outcome of this study was objective response rate (ORR). Secondary outcomes included disease control rate (DCR), progression-free survival (PFS), overall survival (OS) and incidence of treatment-related adverse events (TRAEs). The responses were evaluated by local investigators per Response Evaluation Criteria in Solid Tumors version 1.1. The ORR was defined as the proportion of patients with complete response (CR) or partial response (PR) as their best response. The DCR was defined as the proportion of patients with CR, PR or stable disease (SD). The OS was defined as the time from treatment initiation to death from any cause. The PFS was defined as the time from treatment initiation to the first documented disease progression or death. TRAEs were evaluated according to the Common Terminology Criteria for Adverse Events version 4.0. Baseline characteristics of patients before the treatment initiation were collected for exploratory analyses. The MSI/MMR status was examined in each center by local investigators. The MMR status was determined by immunohistochemistry examining the expression of the four MMR enzymes (MLH1, MSH2, MSH6 and PMS2). The MSI status was determined by polymerase chain reaction assays examining the five microsatellite loci (BAT24, BAT26, D5S346, D2S123 and D17S250). The baseline neutrophil–lymphocyte ratio (NLR) was calculated from the baseline complete blood count (CBC) results if available.

### Statistical analysis

We used R (version 4.0.3) to perform all statistical analyses. Patients were included for efficacy analysis if they had confirmed treatment discontinuation, available radiologic evaluation or at least eight weeks of follow-up after the initiation of study treatment. Patients with at least one available laboratory or vital sign measurement after the study treatment were included for safety analysis. We estimated the OS and PFS using the Kaplan–Meier method. We performed exploratory analyses on DCR and PFS to evaluate the potential effect of clinical variables on responses. The DCR was analyzed using logistic regression model, and the PFS was analyzed using Cox proportional hazard model. Univariate analysis and multivariate analysis adjusting for age, Eastern Cooperative Oncology Group Performance Status (ECOG PS), RAS mutation status and site of primary tumor were performed. The optimal cutoff value of NLR was defined using the maximally selected rank statistics method [22]. The proportional hazard (PH) assumption was tested by the Schoenfeld residuals test. All statistical tests were two-tailed, and *P* < 0.05 was considered statistically significant. The incidence of TRAEs was analyzed in a descriptive method.

## Results

### Patients

A total of 84 patients were identified. Table 1 summarizes the baseline characteristics of patients at treatment initiation. The median age was 63 years (range, 35–81 years). The majority of the patients were male (60%), had ECOG PS of 0 or 1 (98%) and had tumors on the left side (76%). Fifty-nine patients (70%) had multiple metastatic sites; the most common metastatic sites included liver (65%), lung (56%) and lymph node (36%). Forty-five patients (54%) had KRAS or NRAS mutant tumors, and three patients (4%) had BRAF^V600E^ mutations. The MSS/pMMR status was confirmed in 76 patients (90%). Most patients (91%) received two or more systemic treatment lines before the study treatment, and antiangiogenic treatment was previously used in 73 patients (87%).

**Table 1:** Baseline characteristics of included patients (*n* = 84)

Characteristics	No. (%)
No. of patients	84
Median age, years (range)	63 (35–81)
Age, years	
< 70	71 (85)
≥ 70	13 (15)
Male sex	50 (60)
ECOG PS	
0	21 (25)
1	61 (73)
2	2 (2)
Site of primary tumor	
Right-side colon	20 (24)
Left-side colon and rectum	64 (76)
Synchronous metastases	49 (58)
Number of metastatic sites	
Single	25 (30)
Multiple	59 (70)
Site of metastases	
Lymph node	30 (36)
Liver	55 (65)
Lung	47 (56)
Peritoneum	18 (21)
Bone	9 (11)
Mutation status	
BRAF, KRAS, NRAS all wild type	29 (35)
KRAS or NRAS mutant	45 (54)
BRAF^V600E^ mutant ^a^	3 (4)
Unkown	8 (10)
MSS/pMMR status	
Confirmed	76 (90)
Unknown	8 (10)
Median previous systemic treatment lines (range)	3 (0–8)
Prior systemic treatment lines	
0	1 (1)^b^
1	7 (8)
2	25 (30)
3	23 (27)
≥ 4	28 (33)
Prior systemic treatment regimens	
Fluoropyrimidines	82 (98)
Oxaliplatin	72 (86)
Irinotecan	72 (86)
Anti-EGFR treatment	23 (27)
Anti-VEGF treatment	73 (87)
Regorafenib	22 (26)
PD-1 inhibitors	3 (4)
Time from metastatic condition to study treatment initiation	
< 18 months	43 (51)
≥ 18 months	41 (49)
Baseline NLR	
< 1.5	10 (12)
≥ 1.5	72 (86)
Not applicable	2 (2)

^a^One patient has both a BRAF^V600E^ mutation and a NRAS mutation

^b^This patient received adjuvant chemotherapy after surgical resection of the primary tumor

*ECOG PS* Eastern Cooperative Oncology Group Performance Status; *EGFR* epidermal growth factor receptor; *MSS* microsatellite stable; *NLR* neutrophil–lymphocyte ratio; *PD-1* programmed cell death-1; *pMMR* mismatch repair proficient; *VEGF* vascular endothelial growth factor

The characteristics of the study treatment are summarized in Supplementary Table 1. The types of ICIs included sintilimab (39%), nivolumab (20%), toripalimab (15%), camrelizumab (14%), pembrolizumab (7%) and tislelizumab (4%). Among them, sintilimab, toripalimab, camrelizumab and tislelizumab were Chinese domestic ICIs. All ICIs were programmed cell death-1 (PD-1) inhibitors. Most patients (76%) received regorafenib 80 mg as the final dose. At a median follow-up of 5.5 months (95% confidence interval [CI], 4.1–8.5), 15 patients (18%) were still on treatment, while other patients discontinued the treatment because of disease progression (54%), TRAEs (17%) or other reasons (12%). The median cycle of ICIs received was 4 (range, 1–24), and the median treatment duration was 4.3 months (range, 0.5–18.8).

### Efficacy

A total of 82 patients were evaluable for response (Table 2). Two patients were excluded due to the lack of radiologic assessment. Four patients (5%) achieved PR and 37 patients (45%) achieved SD as the best response. Among the four patients achieving PR, the types of ICIs included nivolumab (n = 1), pembrolizumab (n = 1), sintilimab (n = 1) and camrelizumab (n = 1). The median duration of response was 5 months (range, 4.8–17.2), and two patients had ongoing responses at the time of analysis, including one patient with PR for 17.2 months. The median duration of disease control was 6.3 months (range, 0.5–17.2).

**Table 2:** Antitumor activity in evaluable patients (*n* = 82)

	No. (%)
Best response	
Complete response	0
Partial response	4 (5)
Stable disease	37 (45)
Progressive disease	41 (50)
Overall response	4 (5)
Disease control	41 (50)
Median duration of response, months (range)^a^	5.0 (4.8 to 17.2 +)
Median duration of disease control, months (range)^b^	6.3 (0.5 to 17.2 +)

^a^The Kaplan–Meier method for censored data was used to calculate the duration. The plus sign ( +) indicates no progressive disease by the time of the last assessment

^b^Disease control was defined as complete response, partial response and stable disease

**Fig. 1:**
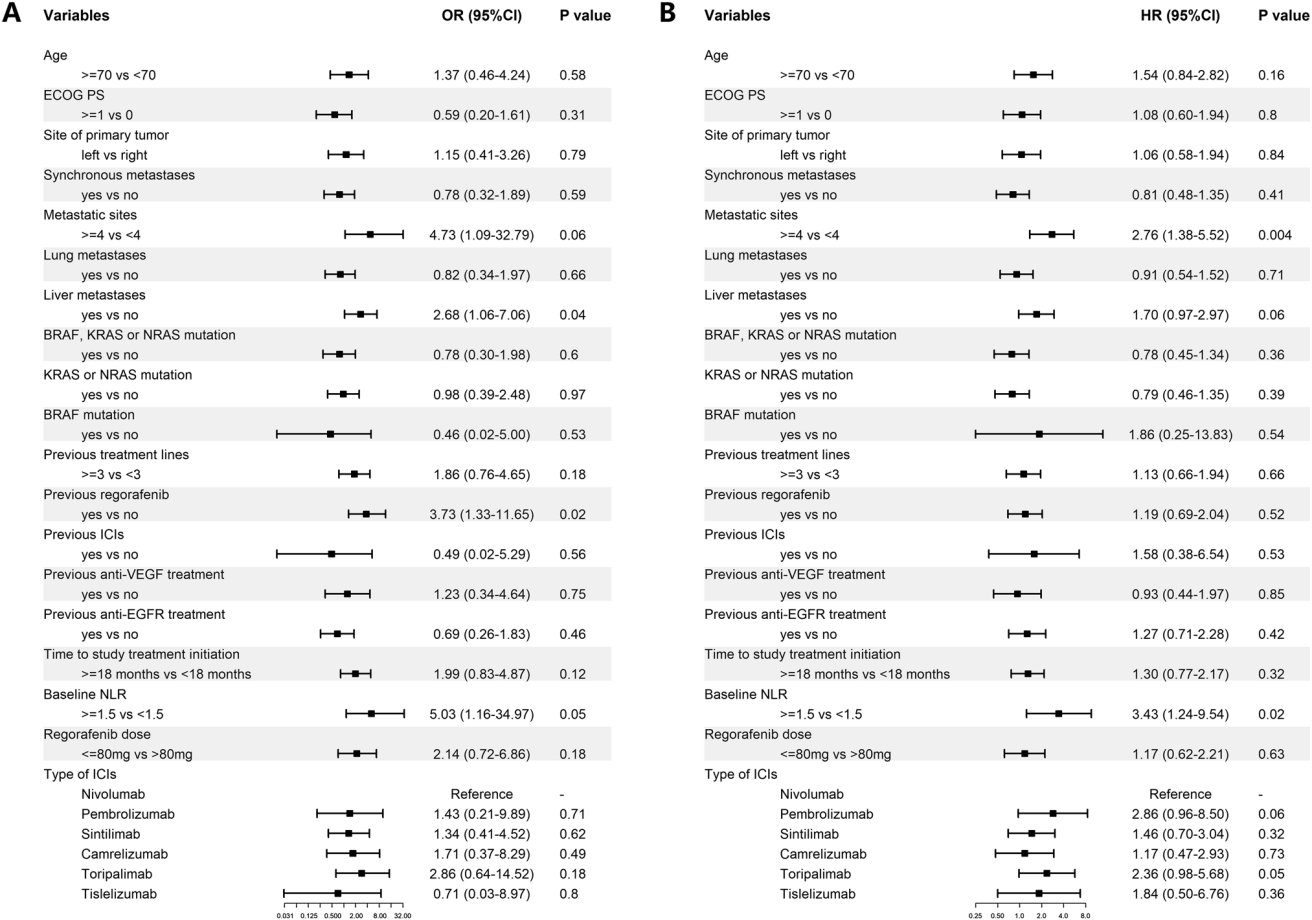
Forest plot of univariate analysis for (**A**) disease control rate and (**B**) progression-free survival *CI* Confidence interval; *ECOG PS* Eastern Cooperative Oncology Group Performance Status; *EGFR* epidermal growth factor receptor; *HR* hazard ratio; *ICI* immune checkpoint inhibitor; *NLR* neutrophil–lymphocyte ratio; *OR* odds ratio; *VEGF* vascular endothelial growth factor

Univariate analysis of DCR revealed that liver metastasis (odds ratio [OR] 2.68, 95% CI 1.06–7.06, P = 0.04), previous regorafenib treatment (OR 3.73, 95%CI 1.33–11.65, P = 0.02) and baseline NLR of ≥ 1.5 (OR 5.03, 95%CI 1.16–34.97, P = 0.05) were associated with increased risk of disease progression (Fig. 1A). After adjusting for age, ECOG PS, RAS mutation status and primary tumor sidedness, the effect of liver metastasis (OR 3.80, 95%CI 1.33–11.76, P = 0.02) and previous regorafenib treatment (OR 3.62, 95%CI 1.12–13.28, P = 0.04) remained statistically significant (Supplementary Table 2). Factors including other previous treatment regimens did not affect the DCR.

**Fig. 2:**
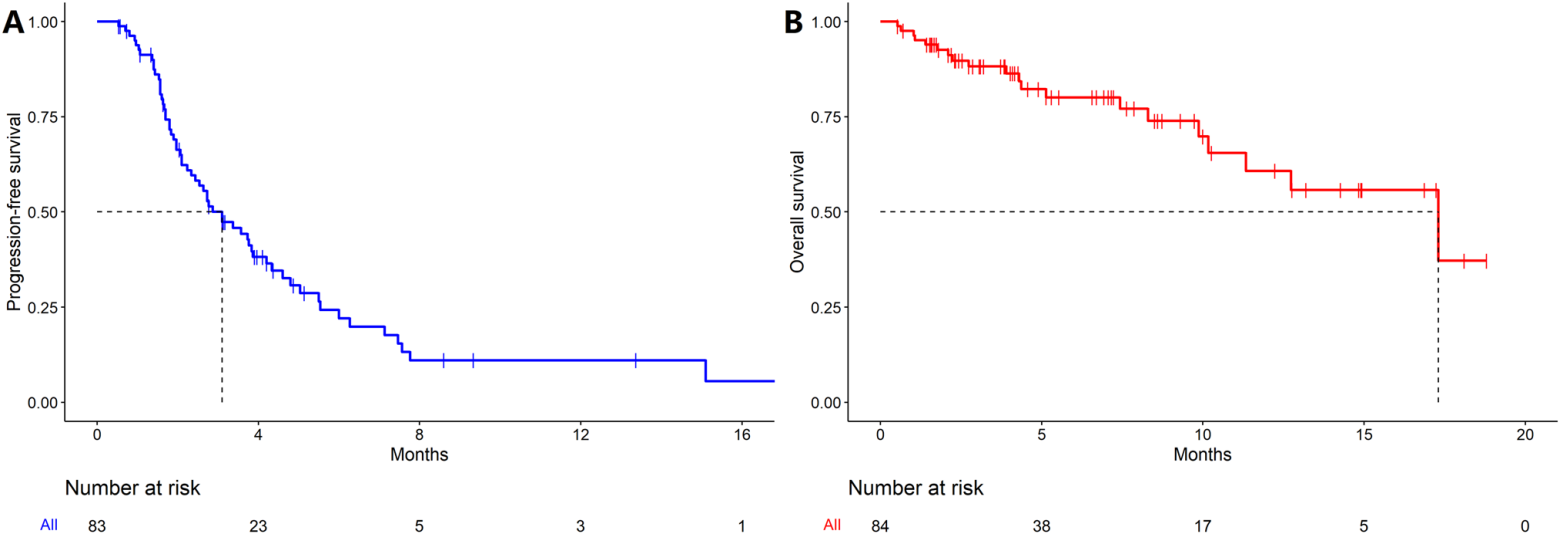
Kaplan–Meier curves of (**A**) progression-free survival (*n* = 83) and (**B**) overall survival (*n* = 84) in patients with colorectal cancer treated with immune checkpoint inhibitors plus regorafenib

All 84 patients were evaluable for OS, and one patient was excluded from PFS analysis due to the lack of radiologic assessment. The median PFS was 3.1 months (95%CI, 2.3–4.2) (Fig. 2A), and the median OS was 17.3 months (95%CI, 11.3—not reached) (Fig. 2B). A total of 29 patients (35%) obtained PFS of ≥ 3 months, and 11 patients (13%) obtained PFS of ≥ 6 months. In the univariate analysis, ≥ 4 metastatic sites (hazard ratio [HR] 2.76, 95%CI 1.38–5.52, P = 0.004) and baseline NLR of ≥ 1.5 (HR 3.43; 95%CI 1.24–9.54, P = 0.02) were associated with shorter PFS (Fig. 1B). Multivariate analysis revealed that ≥ 4 metastatic sites (HR 1.35, 95%CI 1.05–1.73, P = 0.02), liver metastasis (HR 1.98, 95%CI 1.07–3.69, P = 0.03) and baseline NLR of ≥ 1.5 (HR 2.83, 95%CI 1.00–7.98, P = 0.05) were associated with shorter PFS. Other factors, including previous ICIs treatment and previous antiangiogenic treatment, did not affect the PFS.

The PH assumption was satisfied for all clinical variables included in the analysis (Supplementary Table 3).

### Safety

All 84 patients were evaluable for safety. Fifty-one patients (61%) experienced TRAEs during the treatment, and 16 patients (19%) experienced grade 3 TRAEs (Table 3). The most common TRAEs included fatigue (21%), rash (15%) and hand–foot skin reaction (14%); the most common grade 3 TRAEs included rash (7%), hand–foot skin reaction (4%), thrombocytopenia (2%) and myocardial enzyme elevation (2%). One patient experienced treatment-related death because of grade 5 myasthenia gravis. A total of 22 patients (26%) had regorafenib dose reduction or treatment termination because of TRAEs. Among 67 patients who received regorafenib 80 mg or less as the initial dose, 38 patients (57%) experienced TRAEs, including 10 patients (15%) with grade 3 TRAEs; two patients had dose reduction and nine patients discontinued the treatment because of TRAEs. For 17 patients who received regorafenib 120 mg or more as the initial dose, the number of all grade and grade 3 TRAEs were 12 (71%) and five (29%); six patients had dose reduction and five patients discontinued the treatment because of TRAEs. Among 13 patients older than 70 years, eight patients experienced TRAEs (62%), and three patients experienced grade 3 TRAEs (23%).

**Table 3:** Incidence of treatment-related adverse events (*n* = 84)

TRAEs	Any grade, no. (%)	Grade ≥ 3, no. (%)
All	51 (61)	16 (19)
Fatigue	18 (21)	0
Rash	13 (15)	6 (7)
Hand–foot skin reaction	12 (14)	3 (4)
Hypertension	8 (10)	1 (1)
Fever	8 (10)	0
Hypothyroidism	7 (8)	0
Transaminase elevation	7 (8)	0
Diarrhea	6 (7)	0
Anorexia	4 (5)	1 (1)
Oral mucositis	4 (5)	0
Myocardial enzyme elevation	3 (4)	2 (2)
Thrombocytopenia	3 (4)	2 (2)
Hoarseness	3 (4)	0
Myositis	3 (4)	0
Pancreatitis	2 (2)	0
Vomiting	2 (2)	0
Hematuria	1 (1)	1 (1)
Myasthenia gravis	1 (1)	1 (1)
Neutropenia	1 (1)	1 (1)
Visual field loss	1 (1)	1 (1)
Anemia	1 (1)	0
Arthralgia	1 (1)	0
Hyperthyroidism	1 (1)	0
Proteinuria	1 (1)	0

*TRAEs* Treatment-related adverse events

## Discussion

The long-term prognosis of mCRC remains dismal in part because of the lack of effective treatment strategies beyond progression to standard of care [2]. Although recent advances of ICIs have demonstrated remarkable efficacy in patients with MSI-H status, the majority of colorectal cancer patients do not benefit from ICIs due to the immunosuppressive tumor microenvironment of MSS tumors [12, 23]. Currently, many studies are investigating combination strategies to reverse the immunosuppressive microenvironment of MSS colorectal cancer and therefore to exploit the long-term survival benefit of ICIs [12].

In this study, we retrospectively analyzed the efficacy and safety of ICIs combined with regorafenib in patients with MSS colorectal cancer. To our knowledge, this is the largest cohort to evaluate the efficacy and safety of such combination strategy in real-world clinical practice. Our study demonstrated a modest ORR and PFS. Half of the patients could achieve disease control and a proportion of patients could remain progression-free for more than 6 months. This observation corresponded to the long-term survival benefit pattern of ICIs. Conventional treatment options for chemotherapy refractory colorectal cancer included regorafenib single agent and TAS-102 (trifluridine/tipiracil) [2, 24]. For patients whose disease progressed after antiangiogenic treatment, the median PFS was around 2 months and the median OS was 7 months [3, 4, 25]. The results of our study compared favorably with the conventional treatment. Thus, the combination of ICIs with regorafenib could be a feasible treatment option for chemotherapy refractory MSS colorectal cancer.

Several previous studies with small sample sizes have evaluated the efficacy of ICIs combined with regorafenib in MSS colorectal cancer [17, 18, 26]. The results of our study were comparable with the REGOMUNE (NCT03475953) and the REGOTORI study (NCT03946917) [18, 26]. However, in the phase Ib REGONIVO trial (NCT03406871), an ORR of 33% and a DCR of 88% were reported in 24 patients with MSS colorectal cancer, which was higher than the responses reported in other studies [17]. Several factors could account for the difference. First, the types of ICIs were different. The REGONIVO trial only investigated the combination of nivolumab with regorafenib, while patients could receive a variety of ICIs in real-world clinical practice. ICIs other than nivolumab were also analyzed in our study, including several Chinese domestic drugs such as sintilimab and toripalimab. All ICIs were PD-1 inhibitors in this study. Previous studies have suggested superior efficacy of PD-1 inhibitors than programmed cell death ligand-1 (PD-L1) inhibitors, while the difference among different PD-1 inhibitors has not been fully elucidated [27]. Currently, only nivolumab and pembrolizumab have been approved for treating MSI-H colorectal cancer [28]. Although exploratory analyses detected no significant difference in response and PFS among different PD-1 inhibitors in this study, the heterogenous regimens may exhibit different treatment efficacy and require more investigations. Second, the baseline characteristics of patients were different. In the REGONIVO study, all patients had an ECOG PS of 0, and most patients did not bear RAS mutations. These participants represented a subset of patients with good prognosis and they could not reflect the population in routine clinical practice. Third, although the incidence of TRAEs was similar between two studies, only two patients (4%) discontinued the treatment because of TRAEs in the REGONIVO trial, while this number was 14 (17%) in our study. This difference may attribute to the fact that patients in the real world had generally poorer performance status and more comorbidities and requires cautious TRAEs management [29, 30]. However, patients may not receive adequate treatment due to early treatment discontinuation, and thus, the efficacy could be underestimated in the real world. In summary, current evidence has demonstrated promising efficacy of ICIs combined with regorafenib in a subset of patients with MSS colorectal cancer, and further investigations should focus on patient selection.

To help patient selection, we performed exploratory analyses to identify clinical characteristics related to the efficacy of ICIs. Multivariate analysis revealed that liver metastasis was associated with inferior response and PFS. The presence of liver metastasis has been identified as an independent poor prognostic factor for multiple cancer types and particularly for ICIs treatment [31, 32]. More specifically, this association was preserved in clinical trials investigating the combination of regorafenib with ICIs in MSS colorectal cancer. In the REGONIVO trial, compared with the ORR of the entire MSS cohort (33%), only two of 13 patients with liver metastasis (15%) responded to the study treatment [17]. Our result was consistent with previous findings, further confirming the negative predictive role of liver metastasis. The microenvironment of liver metastasis was generally regarded immunosuppressive characterized by decreased infiltration of CD8 + T cells and functional enrichment of immune escape pathways [31, 33, 34]. Moreover, recent studies have revealed that liver metastases could induce systemic resistance to ICIs mediated by macrophages and regulatory T cells [35, 36]. Therefore, effective management of liver metastases could be the key point to overcome the resistance to ICIs.

Our analysis also revealed that a high baseline NLR was associated with inferior PFS. The negative predictive role of NLR has been validated in many studies across different cancer types, and a recent study again confirmed its value in a large cohort of 1714 patients receiving ICIs [37–39]. However, NLR could be affected by factors other than cancer progression, such as infection, steroids use and preexisting autoimmune disease, which limits its value as a cancer predictive biomarker. Besides, current application of NLR lacks a standardized threshold. The cutoff value between the high and the low NLR varied from 1.9 to 7.2 in previous publications and was calculated using certain statistical methods without biological significance [40]. Currently, the dynamic change of peripheral immune cell content during cancer progression and ICIs treatment has not been fully elucidated. Previous studies have suggested that certain neutrophil subsets in the peripheral blood conferred the immunosuppressive ability [41, 42]. Further inspection into the neutrophil heterogeneity may improve the predictive value of NLR. Moreover, for biomarkers other than NLR, a previous study demonstrated superior predictive value of the combination of NLR and TMB than NLR alone [39]. In a small cohort investigating ICIs combined with regorafenib in MSS colorectal cancer, the dynamic change of circulating tumor DNA during early treatment correlated with the response [18]. Predictive models integrating multiple parameters may serve as valuable biomarkers and augment patient selection for the combination strategy.

The toxicity profile of this study was generally tolerable and was comparable with previous studies evaluating the same strategy; the incidence of TRAEs was also similar to conventional treatment such as regorafenib single agent or TAS-102 [3, 4, 17, 18, 26]. Thus, the combination of ICIs with regorafenib could be safely applied in patients with chemotherapy refractory MSS colorectal cancer. Noteworthily, some patients discontinued treatment because of TRAEs. This proportion was similar to the REGOMUNE trial [18]. As what has been mentioned above, early treatment discontinuation may lead to inadequate treatment, and cautiousness should be paid to balance the benefit and risk. Currently, the optimal decision after treatment interruption because of TRAEs remained unclear [43]. In order to fully exploit the benefit of ICIs, retreatment after resolution of TRAEs is a feasible option [44, 45].

This study has several limitations. The major limitation is its retrospective design, which limits the applicability of the results. Second, the median follow-up is relatively short and the OS result remains immature, as half of the events have not occurred at the data cutoff, which may introduce potential confounders to the result. Thus, we only performed exploratory analyses on PFS and DCR to avoid misinterpretations. Third, the MSS status is not available in a small proportion of patients. However, considering that MSI-H/dMMR tumors only account for 2–4% of total mCRC cases, this limitation may not introduce much bias [8].

## Conclusion

In conclusion, the combination of ICIs with regorafenib provides a feasible treatment option for a proportion of patients with chemotherapy refractory MSS colorectal cancer. Prospective validations of this strategy in large cohorts are required, and further inspections into biological rationales may help identify the population who can derive most benefit from this strategy.

### Supplementary Information

Below is the link to the electronic supplementary material.

Supplementary file1 (PDF 182 KB)

## Data Availability

All data are available upon reasonable request.
