# Supplementary material for: Real-world outcomes of regorafenib combined with immune checkpoint inhibitors in patients with advanced or metastatic microsatellite stable colorectal cancer: A multicenter study
Source: Cancer Immunol Immunother. 2021 Oct 24;71(6):1443–51. doi: 10.1007/s00262-021-03083-3 (PMC9123014; doi:10.1007/s00262-021-03083-3)
Supplement: Supplementary file 1 — Supplementary file1 (PDF 182 KB) [file 262_2021_3083_MOESM1_ESM.pdf]

Supplementary Table 1 Overview of the study treatment (n=84)

Supplementary Table 2 Univariate and multivariate analysis for disease control rate

Supplementary Table 3 Univariate and multivariate analysis for progression-free survival

Supplementary Table 1 Overview of the study treatment (n=84)

**Characteristics**	**No. (%)**
Types of ICIs	
Nivolumab	17 (20)
Pembrolizumab	6 (7)
Sintilimab	33 (39)
Camrelizumab	12 (14)
Toripalimab	13 (15)
Tislelizumab	3 (4)
Final dose of regorafenib, mg [a]	
40	2 (2)
60	1 (1)
80	64 (76)
100	1 (1)
120	10 (12)
160	6 (7)
Median cycles of ICIs received (range)	4 (1-24)
Median treatment duration, months (range)	4.3 (0.5-18.8)
Treatment status	
Ongoing	15 (18)
Terminated because of progressive disease	45 (54)
Terminated because of TRAEs	14 (17)
Terminated because of other reasons	10 (12)

a. The treatment dose was presented as the daily dose of the 21 days on/7 days off schedule. For example, if a patient used regorafenib 40mg /80mg qd alternatively in a 21 days on/7 days off manner, the dose was presented as 60mg.

Abbreviations: ICI, immune checkpoint inhibitor; TRAE, treatment-related adverse event

Supplementary Table 2 Univariate and multivariate analysis for disease control rate [a]

**Variables**	**Univariate analysis OR (95%CI)**	**P value for univariate analysis**	**Multivariate analysis OR (95% CI)**	**P value for multivariate analysis**
Age (≥70 vs <70)	1.37 (0.46-4.24)	0.58	-	-
ECOG PS (≥1 vs 0)	0.59 (0.20-1.61)	0.31	-	-
Site of primary tumor (left vs right)	1.15 (0.41-3.26)	0.79	-	-
Synchronous metastases (yes vs no)	0.78 (0.32-1.89)	0.59	0.97 (0.35-2.64)	0.95
Metastatic sites (≥4 vs <4)	4.73 (1.09-32.79)	0.06	3.97 (0.86-28.42)	0.11
Lung metastases (yes vs no)	0.82 (0.34-1.97)	0.66	0.84 (0.32-2.16)	0.71
Liver metastases (yes vs no)	2.68 (1.06-7.06)	0.04	3.80 (1.33-11.76)	0.02
BRAF, KRAS or NRAS mutation (yes vs no)	0.78 (0.30-1.98)	0.60	-	-
KRAS or NRAS mutation (yes vs no)	0.98 (0.39-2.48)	0.97	-	-
BRAF mutation (yes vs no)	0.46 (0.02-5.00)	0.53	-	-
Previous treatment lines (≥3 vs <3)	1.86 (0.76-4.65)	0.18	1.64 (0.62-4.39)	0.32
Previous regorafenib (yes vs no)	3.73 (1.33-11.65)	0.02	3.62 (1.12-13.28)	0.04
Previous ICIs (yes vs no)	0.49 (0.02-5.29)	0.56	0.56 (0.02-6.31)	0.65
Previous anti-VEGF treatment (yes vs no)	1.23 (0.34-4.64)	0.75	1.21 (0.31-4.80)	0.78
Previous anti-EGFR treatment (yes vs no)	0.69 (0.26-1.83)	0.46	0.57 (0.15-2.02)	0.38
Time to study treatment initiation (≥18 months vs <18 months)	1.99 (0.83-4.87)	0.12	2.40 (0.92-6.50)	0.08
Baseline NLR (≥1.5 vs <1.5)	5.03 (1.16-34.97)	0.05	4.30 (0.87-32.32)	0.10
Regorafenib dose (≤80mg vs >80mg)	2.14 (0.72-6.86)	0.18	2.04 (0.62-7.18)	0.25

Type of ICIs				
Nivolumab	Reference	-	Reference	-
Pembrolizumab	1.43 (0.21-9.89)	0.71	1.28 (0.17-9.81)	0.81
Sintilimab	1.34 (0.41-4.52)	0.62	1.11 (0.30-4.24)	0.87
Camrelizumab	1.71 (0.37-8.29)	0.49	1.39 (0.20-10.22)	0.74
Toripalimab	2.86 (0.64-14.52)	0.18	3.91 (0.70-26.62)	0.13
Tislelizumab	0.71 (0.03-8.97)	0.80	0.37 (0.01-5.39)	0.48

a. The multivariate analysis was performed adjusting for the age (≥70), ECOG (≥1), KRAS/NRAS mutation status and site of primary tumor (left) according to the KEYNOTE-177 study.

Abbreviations: CI, confidence interval; ECOG PS, Eastern Cooperative Oncology Group Performance Status; EGFR, epidermal growth factor receptor; ICI, immune checkpoint inhibitor; NLR, neutrophil-lymphocyte ratio; OR, odds ratio; VEGF, vascular endothelial growth factor

Supplementary Table 3 Univariate and multivariate analysis for progression-free survival [a]

Variables	Univariate analysis HR (95%CI)	P value for univariate analysis	Multivariate analysis HR (95% CI)	P value for multivariate analysis	P value for PH assumption
Age (≥70 vs <70)	1.54 (0.84-2.82)	0.16	-	-	0.93
ECOG PS (≥1 vs 0)	1.08 (0.60-1.94)	0.80	-	-	0.37
Site of primary tumor (left vs right)	1.06 (0.58-1.94)	0.84	-	-	0.69
Synchronous metastases (yes vs no)	0.81 (0.48-1.35)	0.41	0.93 (0.51-1.68)	0.80	0.62
Metastatic sites (≥4 vs <4)	2.76 (1.38-5.52)	0.004	1.35 (1.05-1.73)	0.02	0.4
Lung metastases (yes vs no)	0.91 (0.54-1.52)	0.71	1.18 (0.67-2.07)	0.57	0.53
Liver metastases (yes vs no)	1.70 (0.97-2.97)	0.06	1.98 (1.07-3.69)	0.03	0.92
BRAF, KRAS or NRAS mutation (yes vs no)	0.78 (0.45-1.34)	0.36	-	-	0.83
KRAS or NRAS mutation (yes vs no)	0.79 (0.46-1.35)	0.39	-	-	0.8
BRAF mutation (yes vs no)	1.86 (0.25-13.83)	0.54	2.37 (0.31-18.19)	0.41	0.08
Previous treatment lines (≥3 vs <3)	1.13 (0.66-1.94)	0.66	1.04 (0.58-1.86)	0.89	0.79
Previous regorafenib (yes vs no)	1.19 (0.69-2.04)	0.52	1.27 (0.65-2.45)	0.48	0.43
Previous ICIs (yes vs no)	1.58 (0.38-6.54)	0.53	1.33 (0.31-5.69)	0.7	0.09
Previous anti-VEGF treatment (yes vs no)	0.93 (0.44-1.97)	0.85	1.05 (0.48-2.26)	0.91	0.95
Previous anti-EGFR treatment (yes vs no)	1.27 (0.71-2.28)	0.42	1.20 (0.55-2.65)	0.65	0.53
Time to study treatment initiation (≥18 months vs <18 months)	1.30 (0.77-2.17)	0.32	1.23 (0.70-2.18)	0.47	0.82
Baseline NLR (≥1.5 vs <1.5)	3.43 (1.24-9.54)	0.02	2.83 (1.00-7.98)	0.05	0.44

Regorafenib dose (≤80mg vs >80mg)	1.17 (0.62-2.21)	0.63	1.05 (0.52-2.14)	0.89	0.4
Type of ICIs					0.09
Nivolumab	Reference	-	Reference	-	
Pembrolizumab	2.86 (0.96-8.50)	0.06	2.78 (0.88-8.78)	0.08	
Sintilimab	1.46 (0.70-3.04)	0.32	1.05 (0.47-2.37)	0.9	
Camrelizumab	1.17 (0.47-2.93)	0.73	0.85 (0.26-2.78)	0.78	
Toripalimab	2.36 (0.98-5.68)	0.05	1.94 (0.75-5.02)	0.17	
Tislelizumab	1.84 (0.50-6.76)	0.36	1.48 (0.37-5.87)	0.58	

a. The multivariate analysis was performed adjusting for the age (≥70), ECOG (≥1), KRAS/NRAS mutation status and site of primary tumor (left) according to the KEYNOTE-177 study.

Abbreviations: CI, confidence interval; ECOG PS, Eastern Cooperative Oncology Group Performance Status; EGFR, epidermal growth factor receptor; ICI, immune checkpoint inhibitor; HR, hazard ratio; NLR, neutrophil-lymphocyte ratio; PH, proportional hazard; VEGF, vascular endothelial growth factor
